# Shared facial emotion processing functional network findings in medication-naïve major depressive disorder and healthy individuals: detection by sICA

**DOI:** 10.1186/s12888-018-1631-0

**Published:** 2018-04-10

**Authors:** Jian Li, E. Kale Edmiston, Yanqing Tang, Guoguang Fan, Ke Xu, Fei Wang, Jiansong Xu

**Affiliations:** 1 Department of Radiology, The First Hospital of China Medical University, Shenyang, Liaoning People’s Republic of China; 2 Department of Psychiatry, University of Pittsburgh School of Medicine, Pittsburgh, PA USA; 3 Department of Psychiatry, The First Hospital of China Medical University, Shenyang, Liaoning People’s Republic of China; 4 Department of Psychiatry, Yale University School of Medicine, New Haven, CT USA

## Abstract

**Background:**

The fundamental mechanism underlying emotional processing in major depressive disorder (MDD) remains unclear. To better understand the neural correlates of emotional processing in MDD, we investigated the role of multiple functional networks (FNs) during emotional stimuli processing.

**Methods:**

Thirty-two medication-naïve subjects with MDD and 36 healthy controls (HCs) underwent an emotional faces fMRI task that included neutral, happy and fearful expressions. Spatial independent component analysis (sICA) and general linear model (GLM) were conducted to examine the main effect of task condition and group, and two-way interactions of group and task conditions.

**Results:**

In sICA analysis, MDD patients and HCs together showed significant differences in task-related modulations in five FNs across task conditions. One FN mainly involving the ventral medial prefrontal cortex showed lower activation during fearful relative to happy condition. Two FNs mainly involving the bilateral inferior frontal gyrus and temporal cortex, showed opposing modulation relative to the ventral medial prefrontal cortex FN, i.e., greater activation during fearful relative to happy condition. Two remaining FNs involving the fronto-parietal and occipital cortices, showed reduced activation during both fearful and happy conditions relative to the neutral condition. However, MDD and HCs did not show significant differences in expression-related modulations in any FNs in this sample.

**Conclusions:**

SICA revealed differing functional activation patterns than typical GLM-based analyses. The sICA findings demonstrated unique FNs involved in processing happy and fearful facial expressions. Potential differences between MDD and HCs in expression-related FN modulation should be investigated further.

**Electronic supplementary material:**

The online version of this article (10.1186/s12888-018-1631-0) contains supplementary material, which is available to authorized users.

## Background

Major depressive disorder (MDD) is a common and serious mental illness linked to major role impairment, reduced life satisfaction, and high rates of mortality, including suicide [1, 2]. Despite increasing numbers of functional magnetic resonance imaging (fMRI) studies investigating abnormalities in affective processing in MDD, the fundamental mechanism underlying emotional processing in MDD remains unclear and is a major research goal. Abnormal processing of emotional stimuli has been consistently implicated in MDD and is thought to increase risk of disease development, maintenance, and relapse [3–5]. Cognitive models of depression suggest that MDD is characterized by mood congruent emotion processing biases, particularly for negative emotional faces [6, 7]. Studies of facial emotion processing provide important information regarding regional brain function abnormalities in MDD and emotional face stimuli are often used in neuroimaging research in MDD.

Neuroimaging studies have made substantial contributions to the understanding of how facial expressions are processed in humans. Some basic emotions can be most reliably recognized from facial expressions (i.e., fear, disgust, anger, happiness, sadness) [8] and have been shown to be universal in their performance and perception [9]. An extensive neural network of areas has been implicated in emotional face processing [10]. For example, during the processing of fearful faces, there is increased neural activation in the amygdala [11, 12], fusiform gyrus [12, 13], inferior frontal gyrus, superior temporal sulcus (STS) [14], and orbitofrontal cortex [15], and deactivation in the ventral anterior cingulate cortex (ACC) [14] in healthy participants. Most of these areas have also been implicated in MDD [16], there is greater activation in amygdala, fusiform gyrus, inferior parietal lobule, inferior frontal gyrus [10], and lower activation in dorsolateral prefrontal cortex (DLPFC) [17], temporal cortex and insula [5] and inferior frontal gyrus [18]. Meanwhile, processing of happy faces has been associated with increased activation in the fusiform gyrus [19] and medial frontal cortex [20], and deactivation in the temporal cortex [14] in healthy participants. MDD has also been associated with increased activation in medial frontal gyrus, insula, middle temporal gyrus and middle occipital gyrus [10], and reduced activation in temporal cortex and insula [21]. These studies were conducted using an hypothesis-driven approach that assesses for differences within several specific networks or between a few regions of interest; such approaches could be biased by a priori definition of regions or networks and do not allow for a broad, discovery-based investigation of neural networks that may contribute to emotional processing differences.

Current neurobiological models for the pathophysiology of MDD postulate that ventral and dorsal subsystems involving multiple functional networks (FNs) are differentially affected in MDD [22–24]. An imbalanced functional integration of these subsystems may lead to a heightened response to negative information in ventral regions (bottom–up) on the one hand, and a failure to regulate this response through dorsal regions (top–down) on the other [25]. However, no studies to date have investigated emotional processing FNs from the whole-brain level, instead choosing to focus on predefined regions or networks of interest. An exploratory whole brain approach would allow for a more nuanced study of the relative contributions of FNs that may work in opposition to each other during the processing of emotional face stimuli. Furthermore, inconsistencies in the literature make interpretation of the role of FNs in face processing challenging. For example, aberrant responses to happy face stimuli have been shown in more ventral lateral PFC (VLPFC) areas [17, 26], as well as to fearful face stimuli in the insula [18, 27]. The potential function of overlapping regions among different FNs requires further clarification.

Independent component analysis (ICA) is a source-blind separation technique that has become a major analysis tool in fMRI studies [28–30]. Spatial ICA (sICA) assumes that most of the components generated by sICA and fMRI data are consistent in spatial patterns across different studies and populations [31–33]. SICA is more sensitive in detecting task-related and resting state activity changes in fMRI signal than the traditional general linear model-based (GLM) analysis. Because sICA uses a data-driven approach, it can reduce noise in the final analysis by separating artifacts from real fMRI signal. Relevant sICA studies have demonstrated unique findings that are not revealed from a standard GLM approach [29, 34–36]. So far, task-based sICA is limited to several tasks that investigate cognitive domains including attention, working memory, and/or response inhibition, finding that the FNs maintain their general spatial patterns but also show task-related modulations, including changes in spatial extent and strength of internal functional connectivity [36, 37]. However, the modulation of FNs during an emotion face paradigm that includes positive, negative and neutral stimuli is unknown. According to previous findings in studies of emotional processing [7, 10–21], likely relevant brain regions include prefrontal, fronto-parietal, auditory, and visual networks in sICA [38, 39]. Understanding the functions of these FNs will improve our understanding of brain functional organization in health and in the neuropathophysiology of MDD.

In order to better understand the role of different FNs, sICA was utilized to extract FNs from a fMRI dataset acquired during an emotional face paradigm in healthy and MDD participants. We aimed to characterize these FNs in medication-naive individuals with MDD using sICA. We also applied GLM to explore emotion processing networks using an exploratory, whole brain approach. By using both sICA and GLM, we wished to assess differing findings using these methods. We hypothesized that sICA would reveal greater activation in the amygdala, fusiform gyrus when processing negative emotions, and greater activation in the insula when processing positive emotional faces in MDD. We also hypothesized that facial emotion processing would be associated with multiple FNs, including prefrontal, fronto-parietal, auditory, and visual networks.

## Methods

### Participants

The Institutional Review Board of the China Medical University approved our study protocol. The participants provided written informed consent after receiving a detailed description of the study.

We recruited 32 medication-naive patients with MDD ages 19–46 years from the outpatient clinic at the Department of Psychiatry, First Affiliated Hospital of China Medical University and the Mental Health Center of Shenyang. MDD diagnosis was confirmed by two trained psychiatrists using the Structured Clinical Interview for DSM-IV. To be included in our study, individuals with MDD had to fulfill the DSM-IV criteria for MDD, have a current depressive episode, have no comorbid Axis I or II diagnoses, have a score indicating clinically significant depressive symptoms on the 17-item Hamilton Rating Scale for Depression (HAMD-17, at least 24) and Hamilton Anxiety Rating Scale (HAM-A), and have no history of psychopharmacological therapy, electroconvulsive therapy, or psychotherapy.

We recruited 36 healthy controls (HC) from Shenyang, China, via advertisement. The absence of DSM-IV Axis I disorders in controls was confirmed by two independent psychiatrists (L.K. and F.W.) using the Structured Clinical Interview for DSM-IV disorders. Individuals with first-degree family members who had a history of DSM-IV Axis I disorders were excluded.

Additional exclusion criteria for both individuals with MDD and controls were the presence of any MRI contraindications, history of head injury or neurologic disorder, history of drug abuse or dependence, and any concomitant medical disorder. All participants were scanned within 24 h of initial contact with the research team. The participants provided written informed consent after receiving a detailed description of the study. The Institutional Review Board of the China Medical University approved our study protocol.

### Emotional face paradigm

During the fMRI scan, each participant completed an event-related implicit facial emotion task, which has been described previously [40, 41]. Participants viewed faces from the Ekman series depicting negative (fear), positive (happiness) or neutral emotional expressions, and were instructed to press a button to make a male-female determination. In brief, 5 male and 5 female faces were each presented for 2 s with inter-stimulus-intervals of 4, 8 or 12 s. Each of the 3 expressions was shown for each individual, for a total of 30 facial stimuli and a run time of 5 min, 6 s in randomized order. Instructions were presented on the computer at the beginning of each run, and subjects were asked to respond as quickly and accurately as possible. We introduced the viewing instructions before scanning, and performed emotional recognition testing after the scan. Subjects in both groups performed similarly and with high accuracy.

### MRI data acquisition and preprocessing

The fMRI data were acquired using a GE Signa HDX 3.0 T MRI scanner at the First Affiliated Hospital of China Medical University, Shenyang, China. Head motion was minimized with restraining foam pads. We used a standard head coil for radiofrequency transmission and reception of the nuclear magnetic resonance signal. The fMRI images were acquired using a spin–echo planar imaging sequence, parallel to the AC-PC line with the following scan parameters: repetition time 2000 ms, echo time 40 ms, image matrix 64 × 64, field of view 24 × 24 cm^2^, 35 contiguous slices of 3 mm and without gap.

Data were analyzed with Statistical Parametric Mapping (SPM8, www.fil.ion.ucl.ac.uk/spm/software/spm8). The first 10 volumes were deleted. Data preprocessing included slice timing, realignment (motion-corrected), spatial normalization and smoothing. Head motion parameters were computed by estimating translation in each direction and the angular rotation about each axis for each volume. We tested for group differences in head motion by using a two-sample *t* test according to mean framewise displacement Jenkinson measurement [42, 43]. Participants were excluded if their head motion was > 2 mm maximum displacement in any of the x, y or z directions or 2 degrees of any angular motion throughout the course of the scan (no participants were excluded). Spatial normalization was performed using a standard EPI template from the Montreal Neurological Institute (MNI),resampled to 3 × 3 × 3 mm and spatially smoothed with an 8 mm full-width at half-maximum (FWHM) Gaussian kernel.

### SPM analysis

We performed single subject analysis first and compared BOLD signal changes using the contrast of [fearful vs. neutral] and [happy vs. neutral]. The contrast maps from each subject were fed into a second level group analysis (i.e., random effect). We used a two sample t-test in SPM to assess for differences in BOLD signal between groups during fearful and happy face conditions. The results were thresholded at cluster level *p* < 0.05, family-wise error (FWE) corrected for voxel-wise whole brain analysis. Voxel level threshold was set at *p* < 0.01.

### SICA analysis

Group ICA algorithm (GIFT, http://icatb.sourceforge.net/, version1.3 h) was used to extract spatially independent components (ICs) from the whole dataset [44, 45]. Data from all participants from the two groups were concatenated into a single dataset and reduced using two stages of principal component analysis (PCA) [44]. We extracted 75 ICs using Infomax algorithm [46]. The reason for using this high model order ICA is that it generates refined components consistent with known anatomical and functional segmentations of the brain [38, 39, 47–51]. The Infomax algorithm generated a spatial map and a timecourse of BOLD signal changes for each IC. This analysis was repeated 50 times using ICASSO for assessing the repeatability of ICs [52] (Additional file 1: Figure S1). The cluster quality index (Iq) was set greater than 0.9, indicating a highly stable ICA decomposition. Finally, IC timecourses and spatial maps were back-reconstructed for each participant [44, 53, 54].

Two investigators (Xu and Li) visually inspected each IC to separate artifacts from FNs. Our diagnostic criteria for artifacts were consistent with the criteria used by Allen and colleagues [38]. ICs with peak voxels at white matter and/or cerebrospinal fluid (CSF) were diagnosed as artifacts. Small dynamic range of power spectra and/or small ratio of the integral of spectral power below 0.10 Hz to the integral of power between 0.15 and 0.25 Hz were used as indicators of artifacts [38, 55]. For defining brain regions associated with each IC, we used the GIFT one sample *t* test tool to create a group-level *t*-map for each IC. The significance threshold was set at voxel height *p* < 0.001, False-Discovery-Rate (FDR) corrected for multiple comparisons of voxel-wise whole-brain analysis. Additional file 2: Figure S2 and Additional file 3: Table S1 show spatial maps of 32 ICs identified as FNs.

### Assessing task-related modulation over timecourses

To examine task engagement of each IC, the temporal sorting function from GIFT was used to perform a multiple regression analysis between IC timecourse and the design matrix of each participant created during first level SPM analysis. For each IC, this regression generated a beta-weight value for each trial type. An increase in the beta-weight value for one trial type relative to another indicates a greater task-related activation of related IC at the first trial type. SPSS (version 21.0, IBM Corporation, Armonk, NY, USA) general linear model for repeated measures were used to assess the main effect of trial type, group, and the interaction of trial type x group on the beta-weight of 32 ICs (confidence intervals were 95%). The significance threshold of all statistical analyses were set at *p* < 0.05, using FDR to correction of multiple comparisons due to multiple ICs. Additional file 4: Table S2 shows the beta-weight and *p* values of each IC.

## Results

The MDD and control groups did not show significant differences in age, gender, and education. The MDD group exhibited a significant higher score on HAMD-17 and HARS relative to the control group (*p*<0.001, Table 1).

**Table 1:** Demographic and clinical characteristics of participants with major depressive disorder and healthy controls.

	Group; mean ± SD^a^			
	MDD	Control		
Characteristic	*n* = 32	*n* = 36	*t* value	*p* value
Age, yr	27.6 ± 9.7	28.6 ± 9.4	t_66_ = 0.43	0.67
Gender, male:female	16:16	18:18	Χ^2^_1_ = 0.000	> 0.99
Education, yr	12.6 ± 3.2	13.6 ± 2.9	*t*_66_ = 1.37	0.17
HAMD-17 score	28.1 ± 5.1	0.85 ± 1.4	*t* = 29.35	< 0.001
HAM-A score	20.6 ± 8.4	0.61 ± 1.4	*t* = 13.54	< 0.001
Illness duration, mo	12.3 ± 15.2	N/A	–	–

*HAMD* Hamilton Rating Scale for Depression, *HAM-A* Hamilton Anxiety Rating Scale, *MDD* major depressive disorder, *N/A* not applicable, *SD* standard deviation

^a^ Unless otherwise indicated

In sICA, among the 32 ICs classified as FNs, five showed significant main effects of task condition. There was no significant IC for main effect of group, or two-way interaction of group and task conditions. Therefore, we combined HCs and patient data together as one group for describing task-related modulations in IC timecourses as described below.

**Fig. 1:**
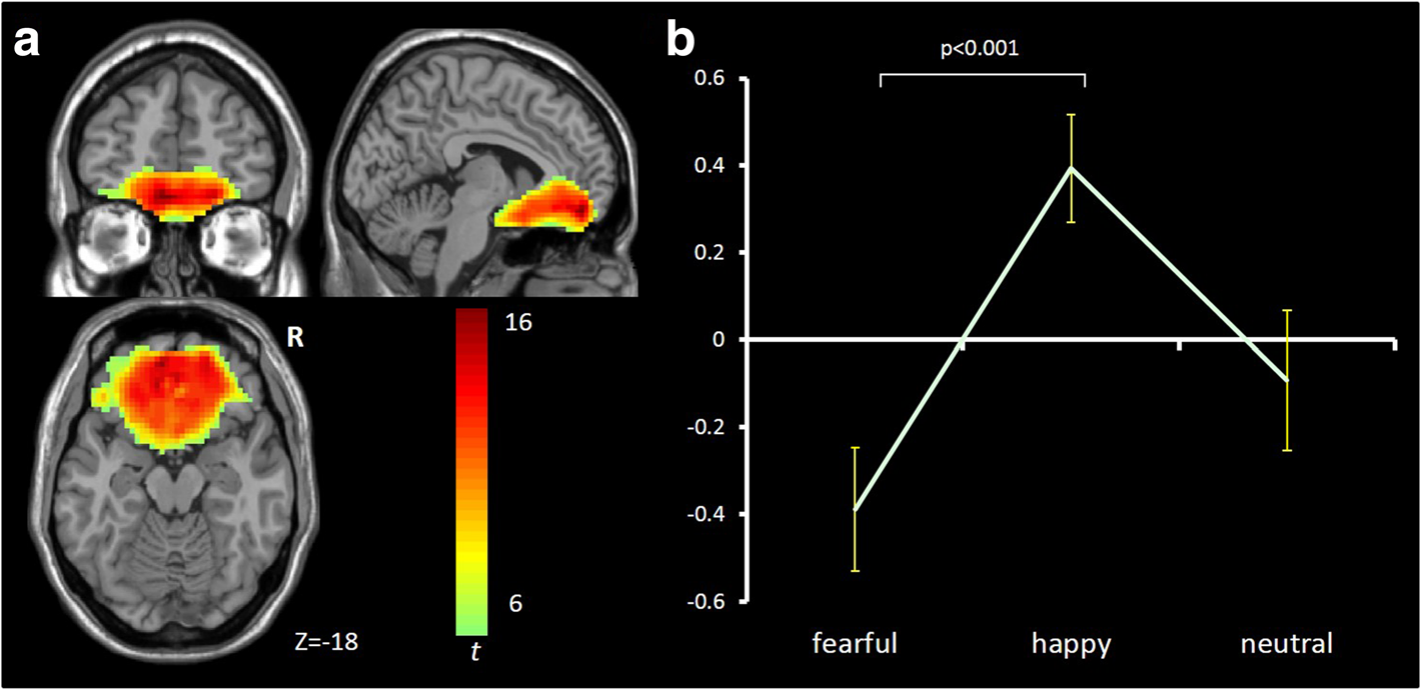
Task-related modulation of IC3 (vmPFC network). **a** Red-yellow color on T1 template in MNI space shows the spatial distribution of IC3. Color bar indicates t values. The number to the bottom right of the axis indicates z coordinates. **b** Line graph shows the beta weight (Y-axis) of IC3 at each task condition (X-axis)

**Fig. 2:**
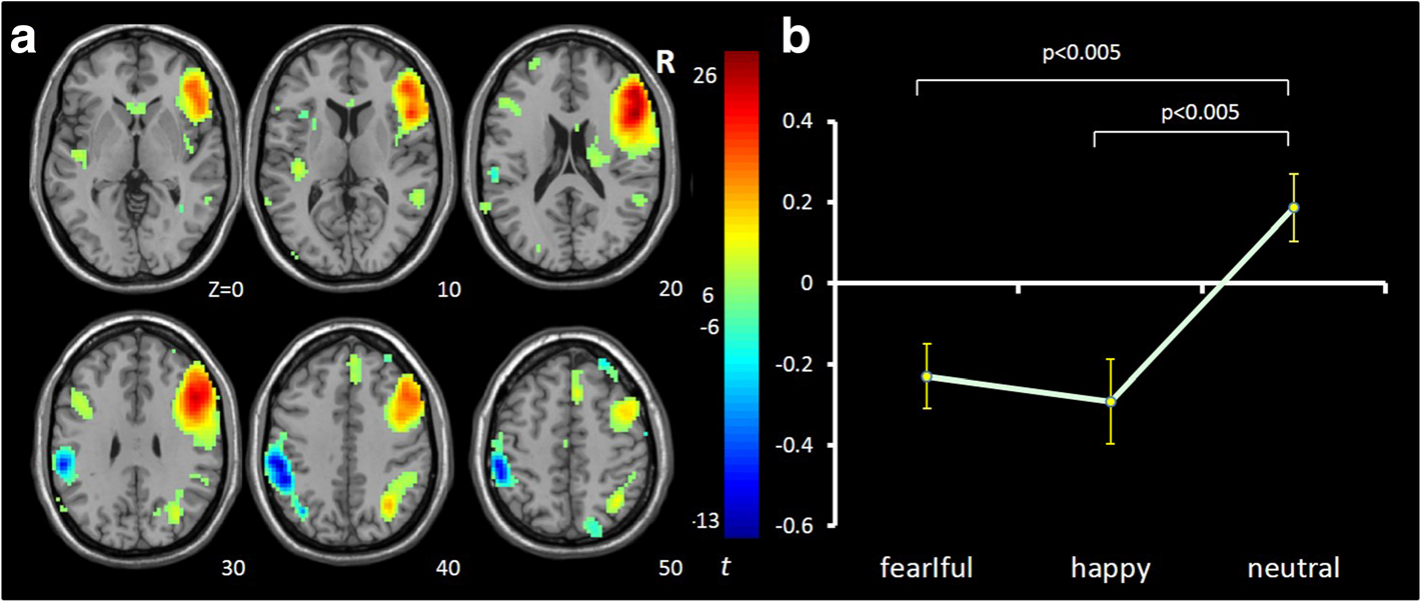
Task-related modulation of IC34 (RFPN). **a** Red-yellow-blue color on T1 template in MNI space shows the spatial distribution of IC34. Color bar indicates t values. The number to the bottom right of the axis indicates z coordinates. **b** Line graph shows the beta weight (Y-axis) of IC34 at each task condition (X-axis)

**Fig. 3:**
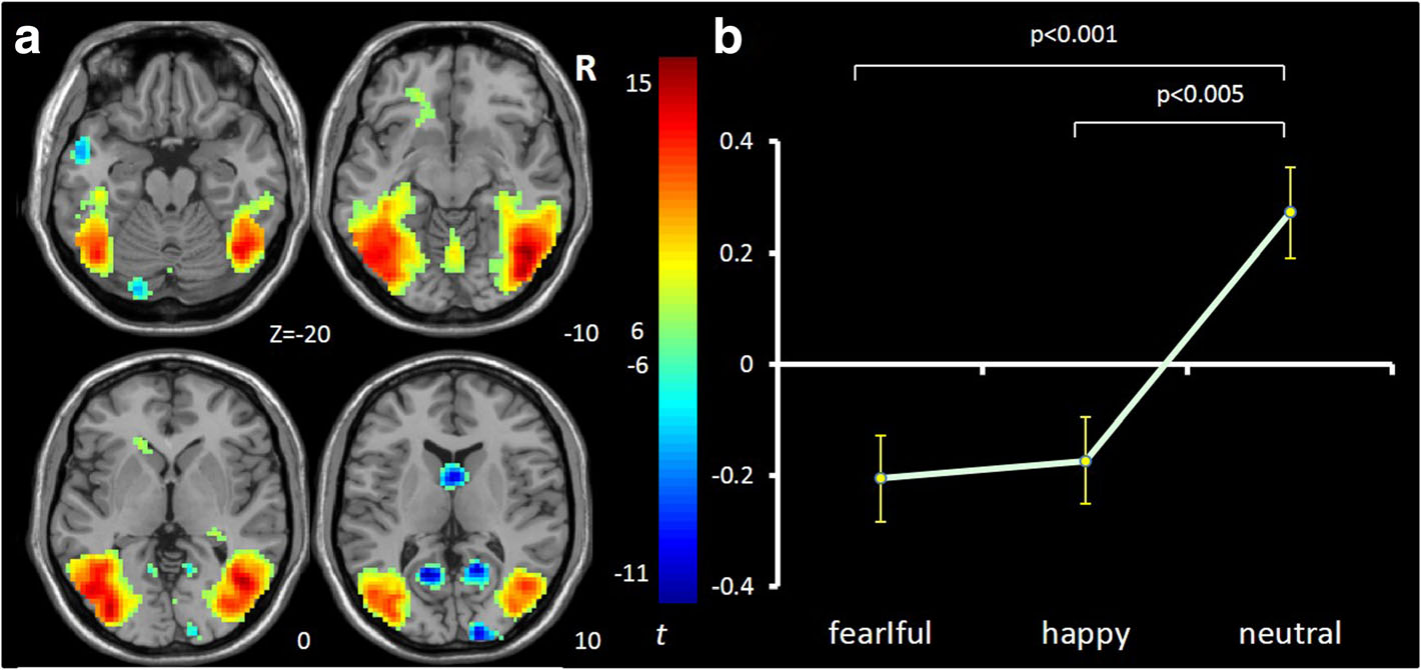
Task-related modulation of IC54 (visual network). **a** Red-yellow-blue color on T1 template in MNI space shows the spatial distribution of IC54. Color bar indicates t values. The number to the bottom right of the axis indicates z coordinates. **b** Line graph shows the beta weight (Y-axis) of IC54 at each task condition (X-axis)

**Fig. 4:**
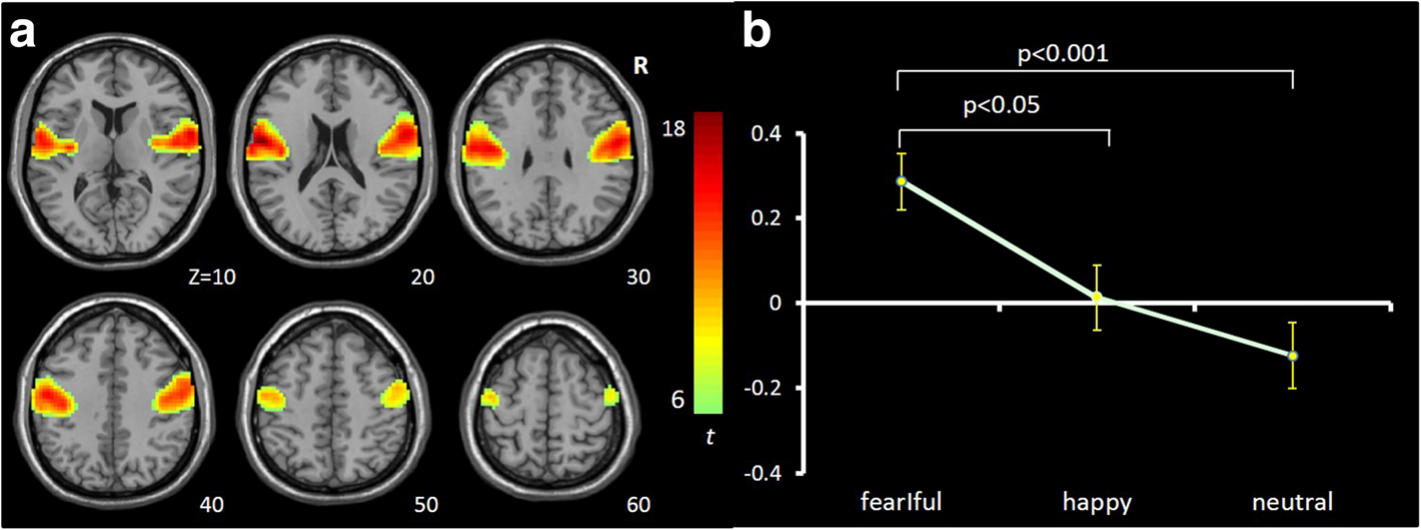
Task-related modulation of IC1 (sensorimotor network). **a** Red-yellow color on T1 template in MNI space shows the spatial distribution of IC1. Color bar indicates t values. The number to the bottom right of the axis indicates z coordinates. **b** Line graph shows the beta weight (Y-axis) of IC1 at each task condition (X-axis)

**Fig. 5:**
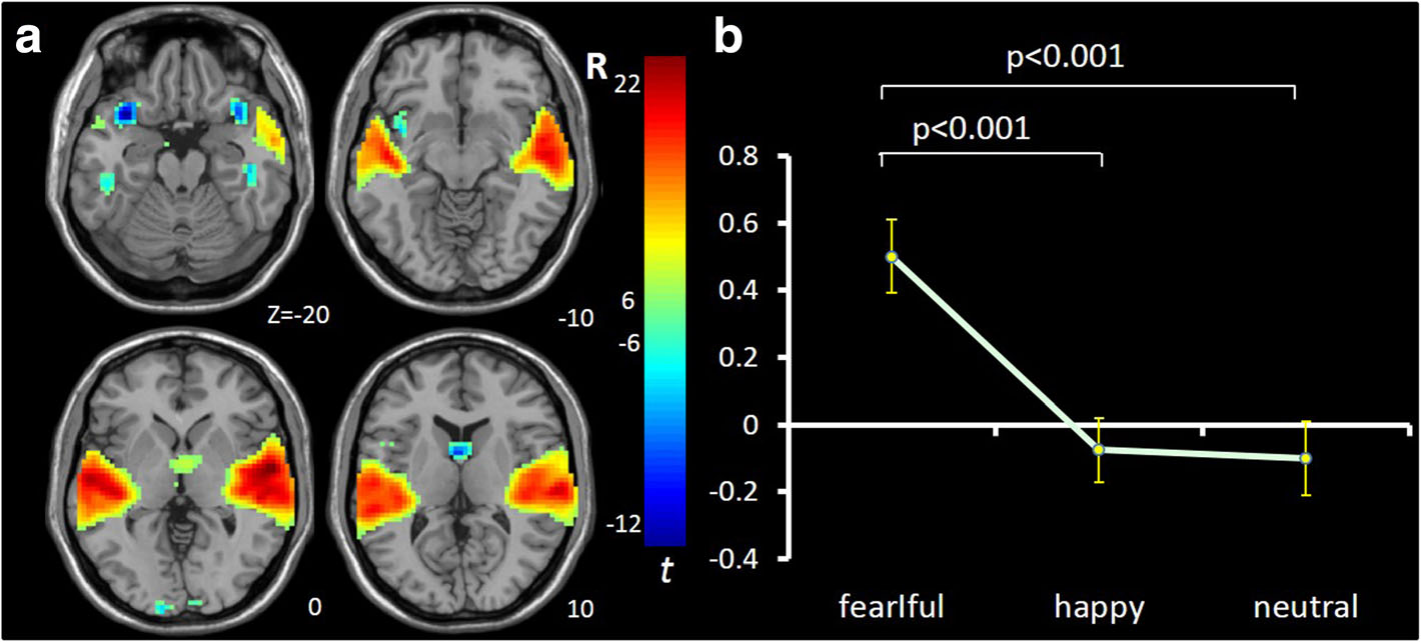
Task-related modulation of IC9 (auditory network). **a** Red-yellow-blue color on T1 template in MNI space shows the spatial distribution of IC9. Color bar indicates t values. The number to the bottom right of the axis indicates z coordinates. **b** Line graph shows the beta weight (Y-axis) of IC9 at each task condition (X-axis)

IC3 [*F*(2,65) = 6.017, *p* = .004, η^2^ = .156] mainly involves ventromedial prefrontal cortex (vmPFC), anterior cingulate cortex (ACC) and bilateral orbital frontal cortex (OFC) and is consistent with the vmPFC network [56–58]. It showed significant opposing modulation between happy vs. fearful faces (Fig. 1). Relative to neutral face, it showed positive and negative modulations during happy and fearful faces, respectively. IC34 [*F*(2,65) = 7.718, *p* = .002, η^2^ = .181] mainly involves the right lateral frontal and parietal cortex and is consistent with the right fronto-parietal network (RFPN) [24, 35, 37, 39, 56]. IC54 [*F*(2,65) = 7.295, *p* = .001, η^2^ = .183] mainly involves the fusiform gyrus and middle occipital gyrus, bilaterally, and is consistent with the visual network [24, 39, 56]. Both of these ICs showed a negative modulation during happy and fearful relative to neutral face conditions (Figs. 2 and 3). IC1 [*F*(2,65) = 8.656, *p* = .000, η^2^ = .210] mainly involves the inferior frontal gyrus (IFG) and precentral gyrus, bilaterally, and is consistent with the sensorimotor network [37, 38, 47]. IC9 [*F*(2,65) = 7.295, *p* = .001, η^2^ = .183] mainly involves the temporal cortex and posterior insula, bilaterally, and is consistent with the auditory network [24, 39, 56]. Both ICs showed positive modulation during fearful relative to happy and neutral faces (Figs. 4 and 5).

We did not observe significant group difference for task conditions, or two-way interaction of group and task conditions in SPM analysis.

## Discussion

To the best of our knowledge, this is the first study to investigate the modulation of FNs during an emotional face task using sICA. SICA is more sensitive in detecting different emotional correlations with the task-positive FNs than GLM, because sICA allows for the detection of both opposing and coincident correlations of fearful and happy conditions, which is not possible in GLM-based analysis. According to our sICA findings, there were significant differences across emotional conditions in 5 ICs, including the vmPFC network, RFPN, visual network, sensorimotor network, and auditory network. The spatial patterns of these FNs are consistent with reported FNs by sICA and fMRI data acquired either during rest or task performance, including a number of visual areas, temporal areas, parietal areas and prefrontal areas [10]. Based on the modulation of emotional face stimuli, the 5 ICs presented were divided into three types. Although we report modulation of multiple FNs, suggesting that extensive neural networks are implicated in face processing, there were not significant differences in diagnostic groups or any two-way interaction effects. This unexpected finding will be discussed in more detail below.

### Fearful<happy

The vmPFC network (IC3) demonstrated a negative correlation with the fearful face condition and a positive correlation with the happy face condition, suggesting opposing modulation of the vmPFC network by emotional valence. As a major region in this network, the vmPFC is known to be involved in emotional processing and affect regulation. The vmPFC has been implicated in human and animal studies of conditioned fear extinction [59, 60]. Coupling between the vmPFC and the amygdala has previously been found during down-regulation of negative affect in healthy controls [61], as reflected by decreasing amygdala activation with increasing vmPFC activation. The inverse functional coupling suggests that vmPFC exerts a top-down inhibitory influence on the activity of the amygdala to transform and down-regulate the experience of negative stimuli [61]. In a similar study in depression, MDD patients showed a positive association between the vmPFC and the amygdala, potentially reflecting impaired top-down control over amygdala responses and inability to down-regulate negative affect [62]. On the other hand, the vmPFC activity is associated with happy faces positively. Previous research has shown that both younger and older adults demonstrate greater vmPFC activity to happy compared to angry or neutral faces [63]. There is evidence that patient with MDD process and interpret positive stimuli as negative [64]. These results suggest different roles for aberrant vmPFC activity in response to negative and positive valence stimuli.

### Fearful and happy<neutral

The RFPN (IC34) and visual network (IC54) demonstrated negative correlation with fearful and happy conditions, and were significant decreased in fearful and happy relative to neutral face conditions. To our knowledge, the overlapping modulation of these two networks in fearful and happy face conditions has not been reported before.

The FPN is commonly engaged in supporting memory, attention, cognitive control and decision-making processes [65]. The laterality of FPN is likely related to different aspects in regulation of emotional processing. Disparate behavior of these lateralized networks has been noted previously [31, 39] and corresponds well with task-based findings. The LFPN is more implicated in explicit memory retrieval related with recollection, whereas the RFPN is likely to be influenced by familiarity [66]. Although the FPN comprises both frontal and parietal regions, most studies have primarily investigated the DLPFC, mainly because the DLPFC plays an important role in the top-down regulation of emotional processing [67–69]. DLPFC has been associated with cognitive or executive functions [70], as well as emotion [71, 72]. Our findings of down modulation for both fearful and happy conditions suggests the RPFN participates in the regulation of emotion generally.

The visual network has been implicated in the perceptual processing of facial expressions [7], including primary, lateral and medial visual cortex [24]. In particular, the face-selective areas in the fusiform gyrus exhibit greater activation to emotional than to neutral faces in both PET and fMRI studies [73, 74], this phenomenon is more common for fearful faces than for happy faces [75, 76]. In the current study, there was similar modulation patterns of the visual network in the processing of both emotional face conditions. These results would be consistent with the existence of direct connections between visual cortex and the amygdala, direct feedback signals from the amygdala [77]. An early mechanism for identifying emotional stimuli would be adaptive; rapid identification and processing of emotional stimuli can signify both potential threat and potential reward [78]. However, we found similar modulation in response to positive and negative emotional stimuli in current study. The unusual finding could be derived from the potential function of overlapping regions within visual network. Whether the mechanism of modulation in positive emotional face stimuli is the same as in negative stimuli warrants further exploration.

### Fearful>happy

The sensorimotor network (IC1) and auditory network (IC9) showed significantly increased activation in the fearful relative to happy and neutral conditions. There was no significant difference between the happy and neutral conditions. Previously, these two networks have not been considered key components concerning emotional stimuli processing and have been given little attention.

The available evidence suggests a role of the IFG in general inhibitory processes [79], and an index of successful inhibition of distracting emotional stimuli in HCs [80, 81]. Emotional distraction often interferes with cognitive processing. Several fMRI studies [82–84] suggest that during attention tasks, enhanced IFG response is associated with the impaired ability to divert attention from task-irrelevant negative emotional information in MDD. We observed that the bilateral IFG within the sensorimotor network showed greater activation in the fearful relative to happy and neutral conditions. Our findings regarding negative emotional stimuli are in line with previous studies.

Early perceptual processing of faces has been suggested to draw on the temporal cortex that construct detailed representations from the configuration of facial features [9], although the exact functional interplay between these areas is not clear. Our findings in the auditory network showed significant activation in response to fearful stimuli. However, deactivation during viewing of fearful faces has been found in the bilateral temporal cortex in adolescents [14]. In another study, whole-brain analyses revealed lower activation in that right superior/middle temporal gyrus in MDD when viewing fearful faces [5]. More research is needed to determine whether such inconsistencies that may be related to differences in subjects, task design, or analytic methodology. The mechanism underlying differences in network modulation requires further investigation.

#### Negative findings

There was no significant IC for main effect of group, or two-way interaction of group and task conditions in this study, which may be ascribed to several possible factors. Unlike many previous studies, all participants with MDD in the present study were experiencing acute depressive episodes; therefore our findings may not necessarily be generalized to the entire MDD population. Future studies of participants in the euthymic state or individuals at risk for MDD are warranted to elucidate connectivity abnormalities associated with MDD neuropathophysiology more generally. Likewise, previous studies have typically included participants who have experienced multiple depressive episodes and therapies for depression, including pharmacological therapies. It could be that our negative finding is due to the fact that our participants were unmedicated, first episode, and had never received treatment of any kind for depression. A previous longitudinal study of duloxetine treatment in MDD found that treatment normalized resting state activity relative to healthy controls, but that there were no differences in activity during an emotional face task at baseline or group x time effects of treatment [85]. Other longitudinal studies of medication effects have found activity differences in the amygdala in response to emotional faces; however, these studies are not of first episode populations [86]. It is likely that our sample includes individuals that will differ in their clinical outcome; some may remit and some may have a more chronic illness course. Future studies are needed to conduct longitudinal assessments of treatment and illness course impact on FN modulation during emotion processing. Additionally, fearful faces were included as a negative emotion in the study. The most frequently observed negative emotional biases in MDD are toward sad and/or angry faces. Future studies including more negative emotions are necessary to better understand the neural correlates of emotion processing in MDD. Negative findings could also be due to the scan duration used here (one run),which might be insufficient to detect significant FN differences. Future studies including more runs and more emotional conditions are needed to fully understand the neuropathophysiology of MDD.

#### Limitations

All participants in this study simply watched the emotional stimuli passively without being asked to respond; no behavioral data (subjective ratings of the emotional value of the stimuli) were collected during the fMRI session. Although passive viewing tasks are useful for engaging implicit emotional processing circuity, passive viewing makes it difficult to control for possible differences in attention or emotion recognition accuracy between the two groups, although we introduced viewing instructions before scanning, and performed emotional recognition testing after the scan that did not show group differences. Subjects in both groups performed similarly and with high accuracy. Additionally, Western faces from the Ekman’s emotional expressions were viewed by participants in emotion paradigm. Although facial expressions of primary emotions are universal, cross-cultural differences in emotion expression intensity could have influenced the way in which participants processed these stimuli, particularly negative emotions. Assessing the validity in Han Chinese populations of standardized emotional face stimuli designed with an assumed Western audience is an important goal for future studies. Finally, although performed using an exploratory approach, sICA is still associated with methodological constraints. The spatial pattern of each IC may be differ dependent on the numbers of ICs extracted. Therefore, the number and location of FN overlap may change based on the number of extracted ICs. Because of this, we have to infer each network’s modularity and hierarchical order based on existing knowledge and available literature. However, it has been demonstrated that ICs remain accurate across a large range of numbers of selected ICs, and many ICs generated by sICA and fMRI data are very consistent in spatial patterns across different studies and populations. Network homogeneity provides a potential alternative approach to assess the homogeneity of specific large-scale networks, which should be used to validate the results in the future.

## Conclusion

This is the first study to investigate the modulation of FNs during implicit processing of face emotions using sICA. Our results demonstrated unique FNs involved in processing happy and fearful facial expressions. These findings suggest that the sICA method may be more sensitive than a GLM approach; sICA is an important complementary tool for assessment of FN modulation, in addition to the GLM method. Although we did not report differences between patients with MDD and controls in this study, future research should assess for effects of mood state, treatment history, and illness course on the emotional modulation of FNs.

## Additional files

Additional file 1: **Figure S1.** ICASSO results. (PDF 125 kb)

Additional file 2: **Figure S2.** Matched ICs from different datasets. (PDF 2121 kb)

Additional file 3: **Table S1.** Brain regions showing task-related changes in BOLD signal assessed by SPM8. (PDF 106 kb)

Additional file 4: **Table S2.** Betaweights at each task condition and related *p* values. (PDF 99 kb)

## Abbreviations

ACC: anterior cingulate cortex
CSF: cerebrospinal fluid
DLPFC: dorsolateral prefrontal cortex
FDR: False-Discovery-Rate
fMRI: functional MRI
FNs: functional networks
FWE: family-wise error
FWHM: full-width at half-maximum
GLM: general linear model
HAM-A: Hamilton Anxiety Rating Scale
HAMD: Hamilton Rating Scale for Depression
HCs: healthy controls
ICA: Independent component analysis
ICs: independent components
IFG: inferior frontal gyrus
Iq: quality index
MDD: major depressive disorder
MNI: Montreal Neurological Institute
N/A: not applicable
OFC: orbital frontal cortex
PCA: principal component analysis
RFPN: right fronto-parietal network
SD: standard deviation
sICA: Spatial independent component analysis
VLPFC: ventral lateral PFC
vmPFC: ventromedial prefrontal cortex
