# Supplementary material for: Shared facial emotion processing functional network findings in medication-naïve major depressive disorder and healthy individuals: detection by sICA
Source: BMC Psychiatry. 2018 Apr 10;18:96. doi: 10.1186/s12888-018-1631-0 (PMC5891939; doi:10.1186/s12888-018-1631-0)
Supplement: Supplementary file 1 — Figure S1. ICASSO results. (PDF 125 kb) [file 12888_2018_1631_MOESM1_ESM.pdf]

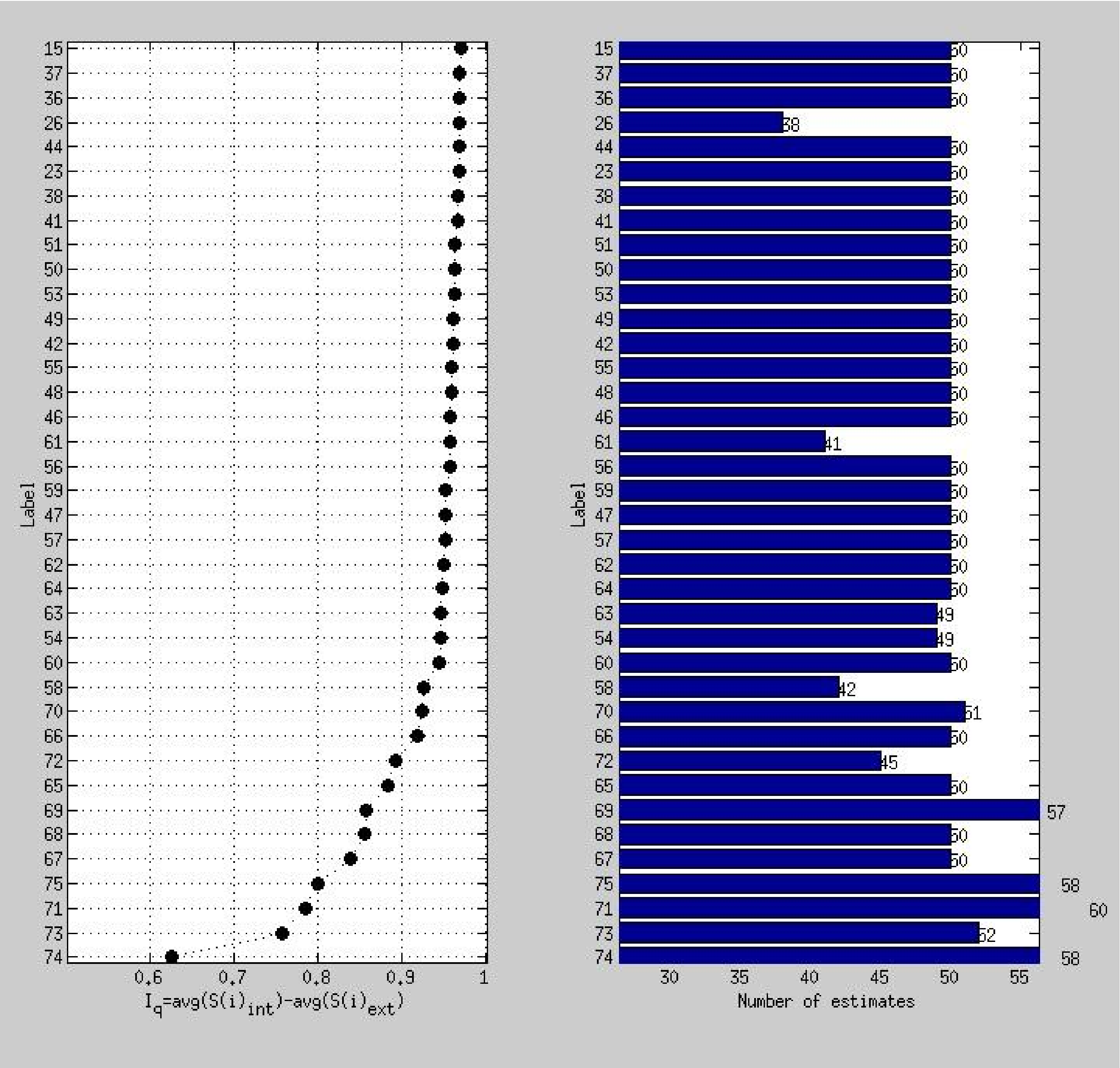

Figure S1: ICASSO results: Stability quality index (Iq) and number of ICA estimates in the estimate-clusters. The stability index of each component was greater than 0.90.
