# Supplementary material for: Shared facial emotion processing functional network findings in medication-naïve major depressive disorder and healthy individuals: detection by sICA
Source: BMC Psychiatry. 2018 Apr 10;18:96. doi: 10.1186/s12888-018-1631-0 (PMC5891939; doi:10.1186/s12888-018-1631-0)
Supplement: Supplementary file 2 — Figure S2. Matched ICs from different datasets. (PDF 2121 kb) [file 12888_2018_1631_MOESM2_ESM.pdf]

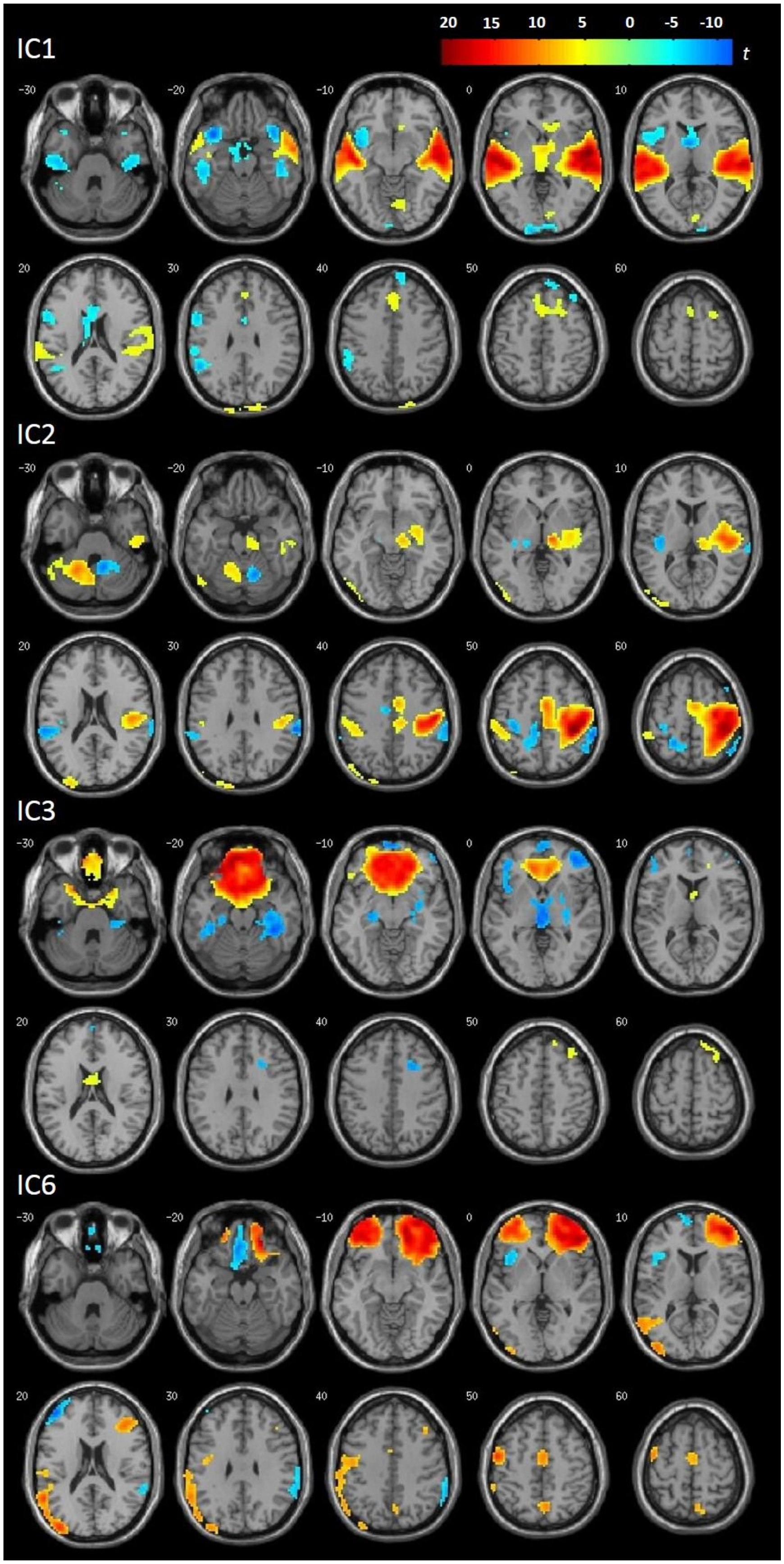

20 15 10 5 0 -5 -10
t
IC1
IC2
IC3
IC6
-30
-20
-10
0
10
20
30
40
50
60

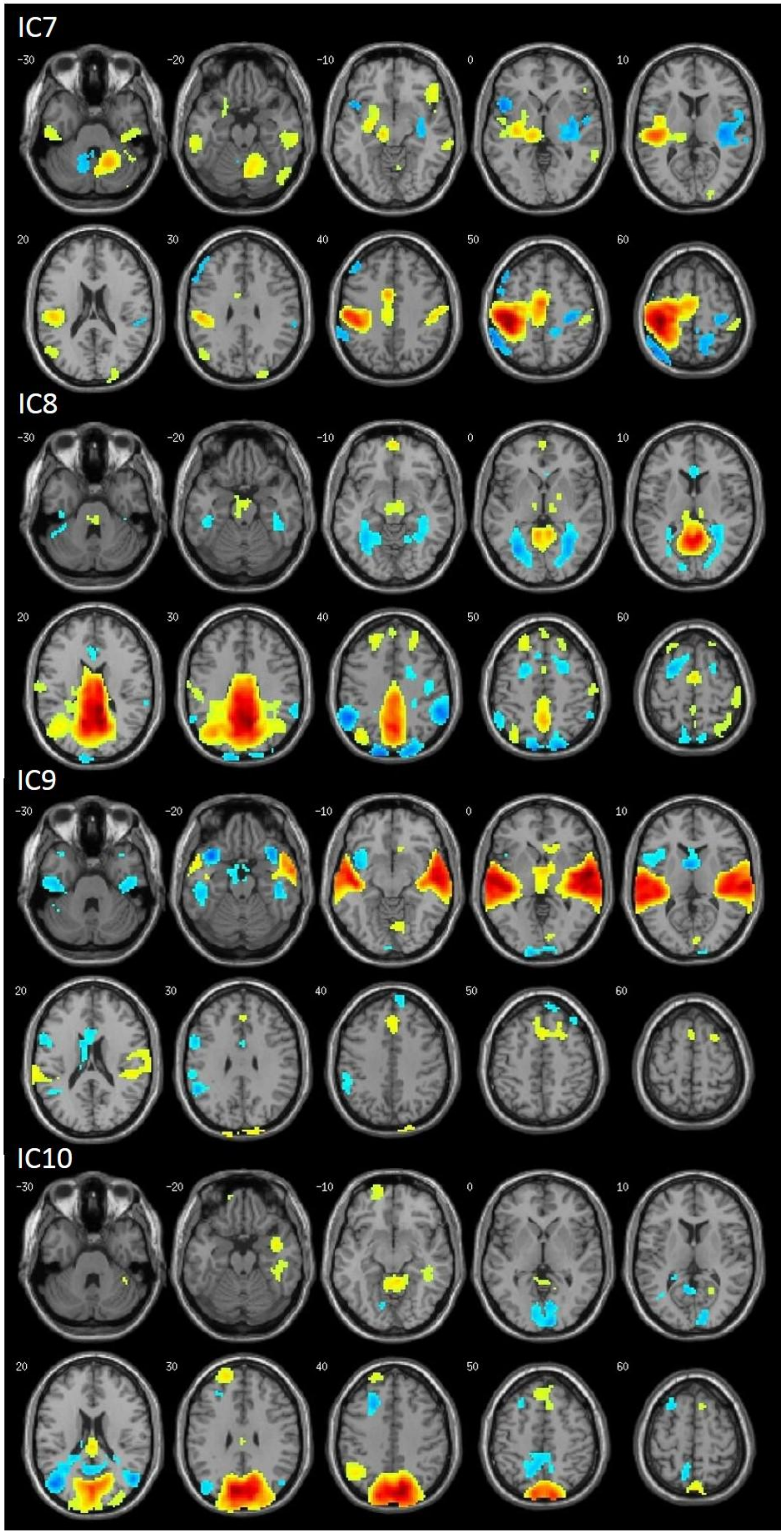
IC7
-30
-20
-10
0
10
20
30
40
50
60
IC8
-30
-20
-10
0
10
20
30
40
50
60
IC9
-30
-20
-10
0
10
20
30
40
50
60
IC10
-30
-20
-10
0
10
20
30
40
50
60

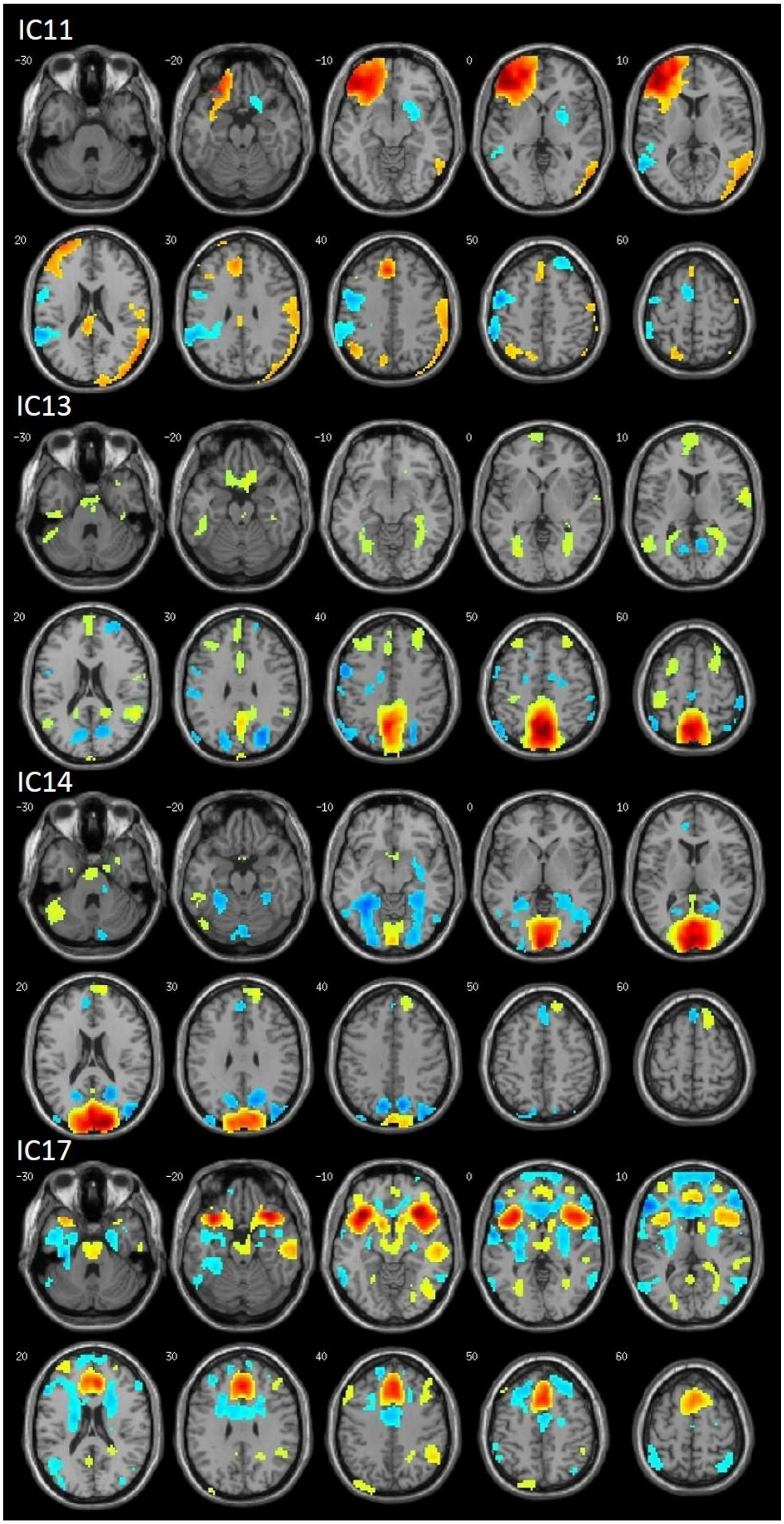
IC11
IC13
IC14
IC17

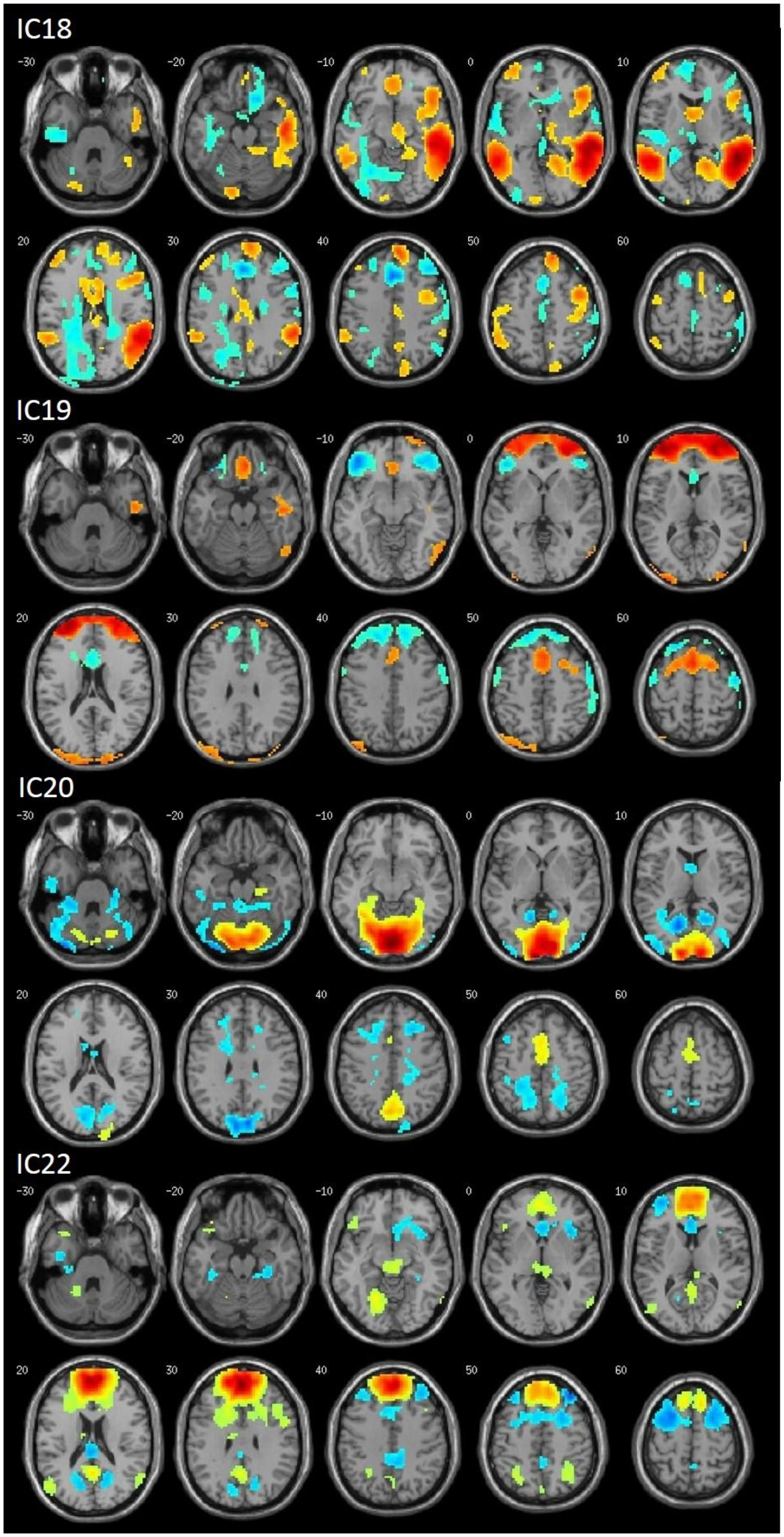
IC18
-30
-20
-10
0
10
20
30
40
50
60
IC19
-30
-20
-10
0
10
20
30
40
50
60
IC20
-30
-20
-10
0
10
20
30
40
50
60
IC22
-30
-20
-10
0
10
20
30
40
50
60

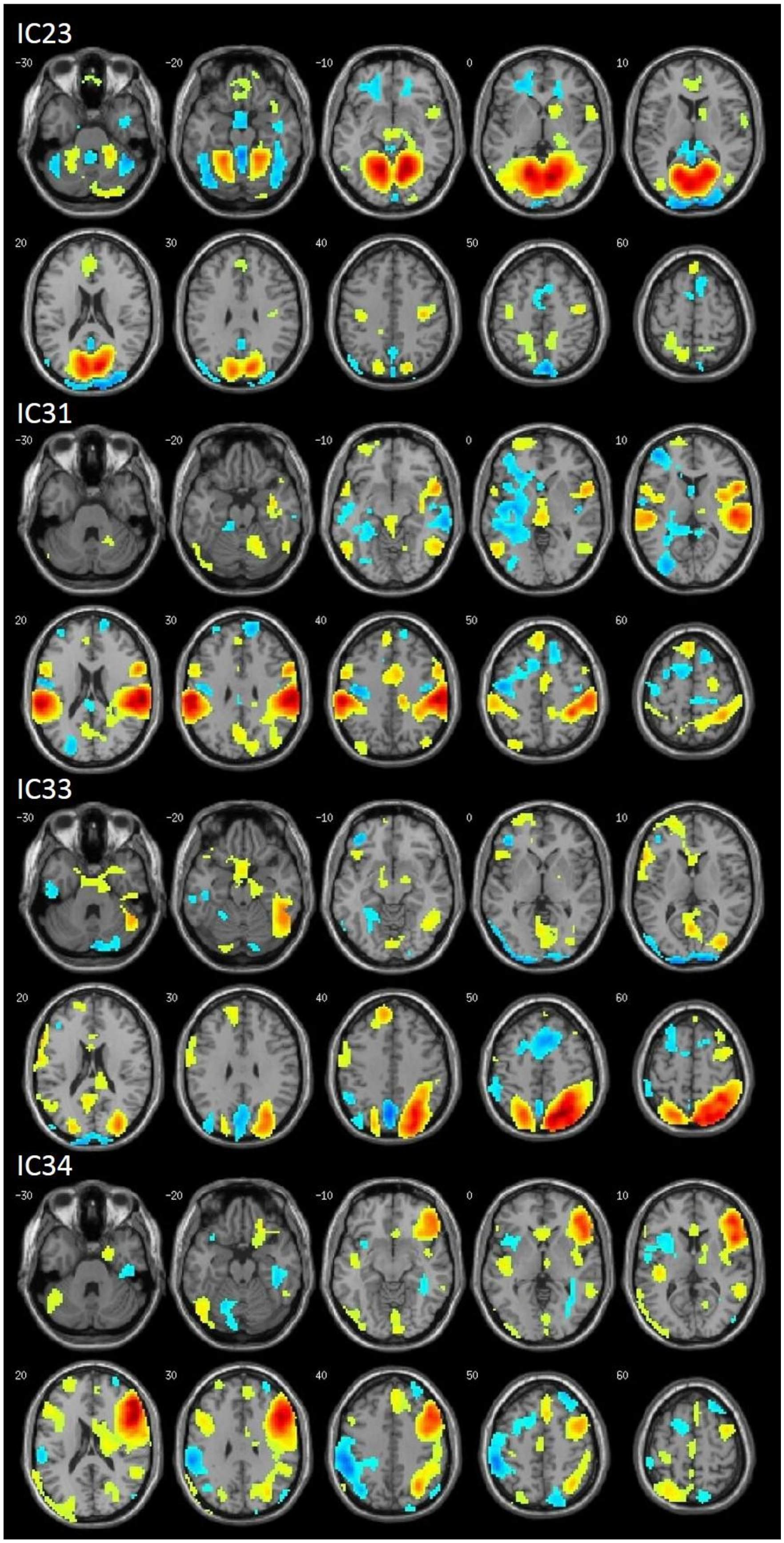
IC23
-30
-20
-10
0
10
20
30
40
50
60
IC31
-30
-20
-10
0
10
20
30
40
50
60
IC33
-30
-20
-10
0
10
20
30
40
50
60
IC34
-30
-20
-10
0
10
20
30
40
50
60

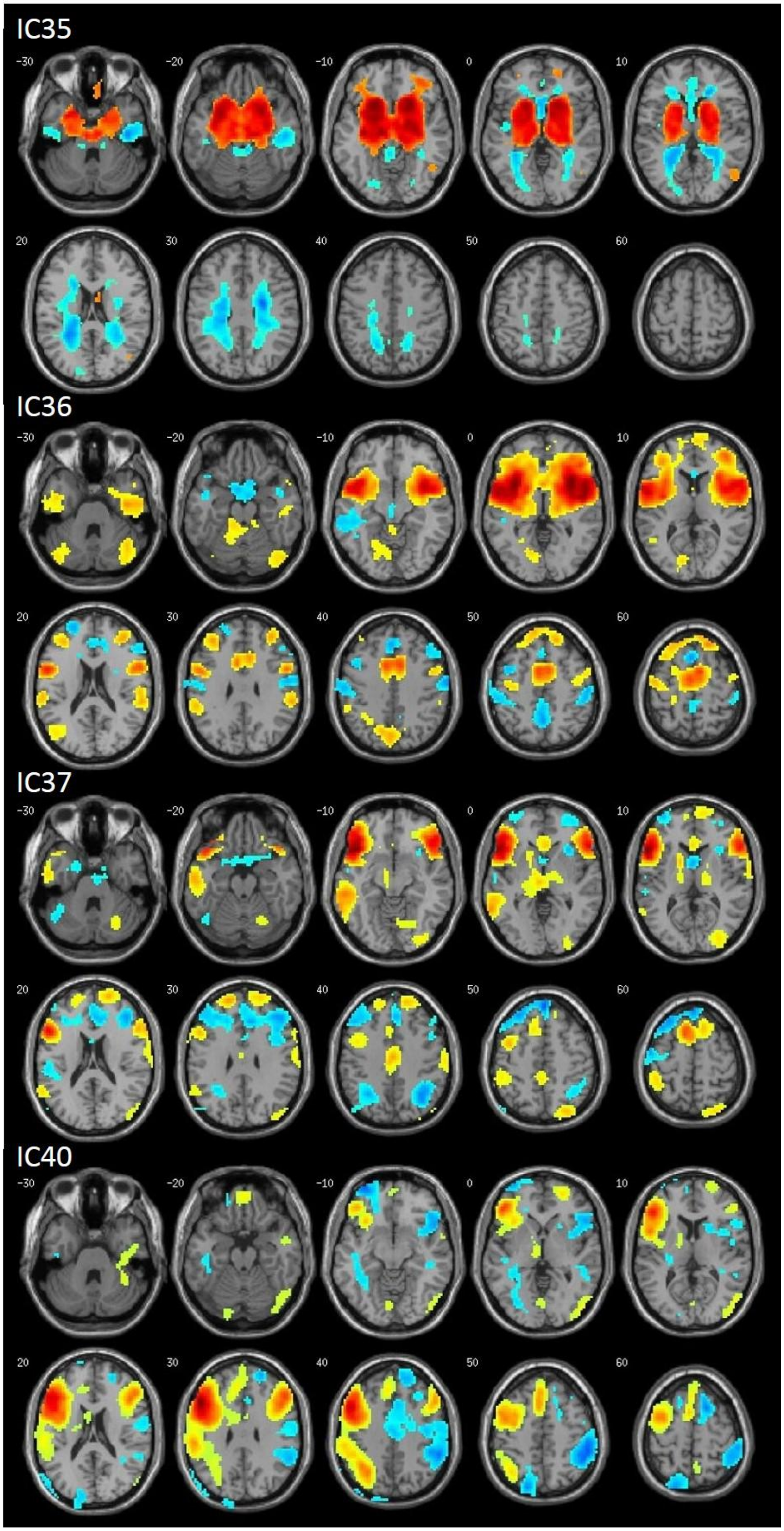
IC35
-30
-20
-10
0
10
20
30
40
50
60
IC36
IC37
IC40

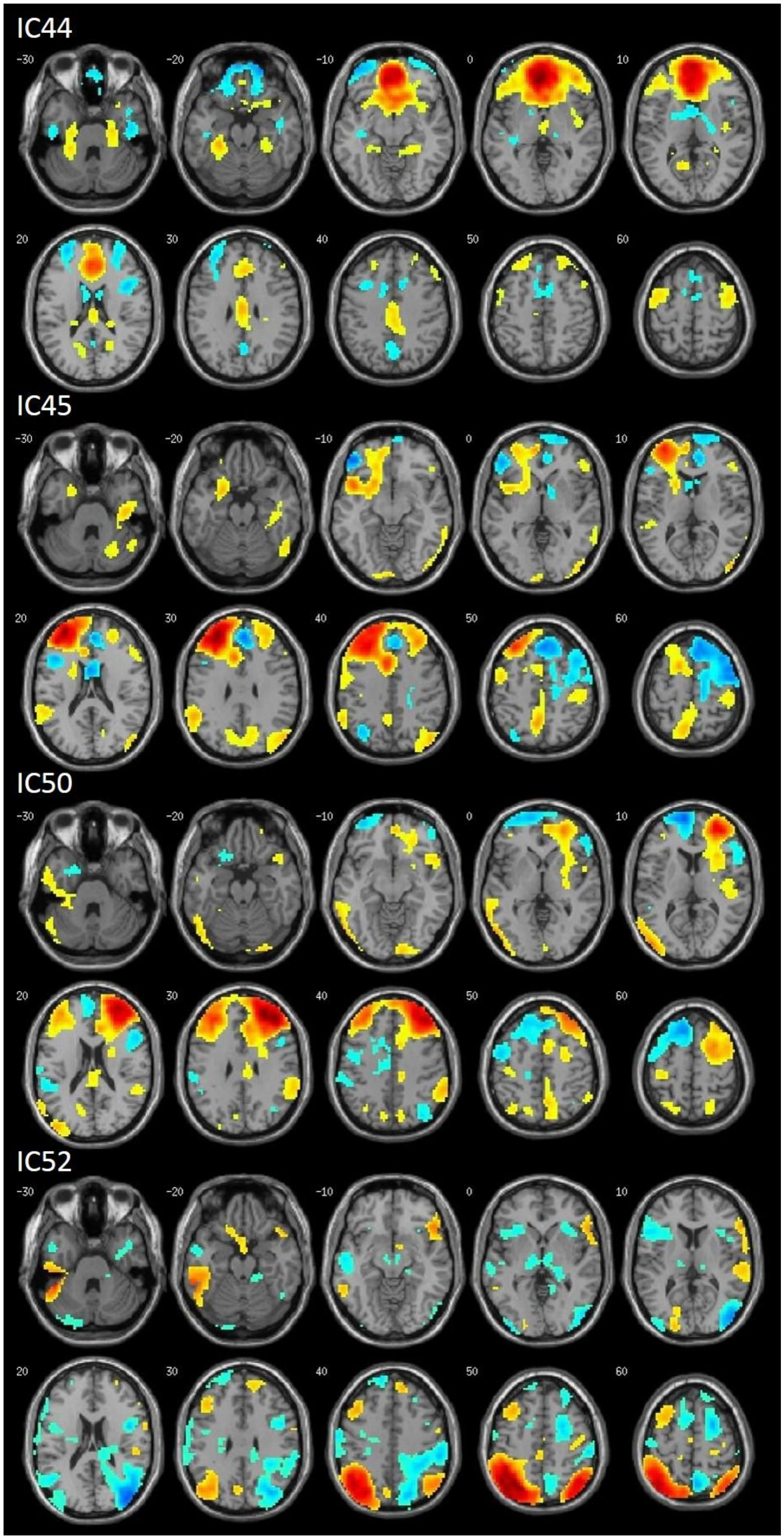
IC44
-30
-20
-10
0
10
20
30
40
50
60
IC45
IC50
IC52

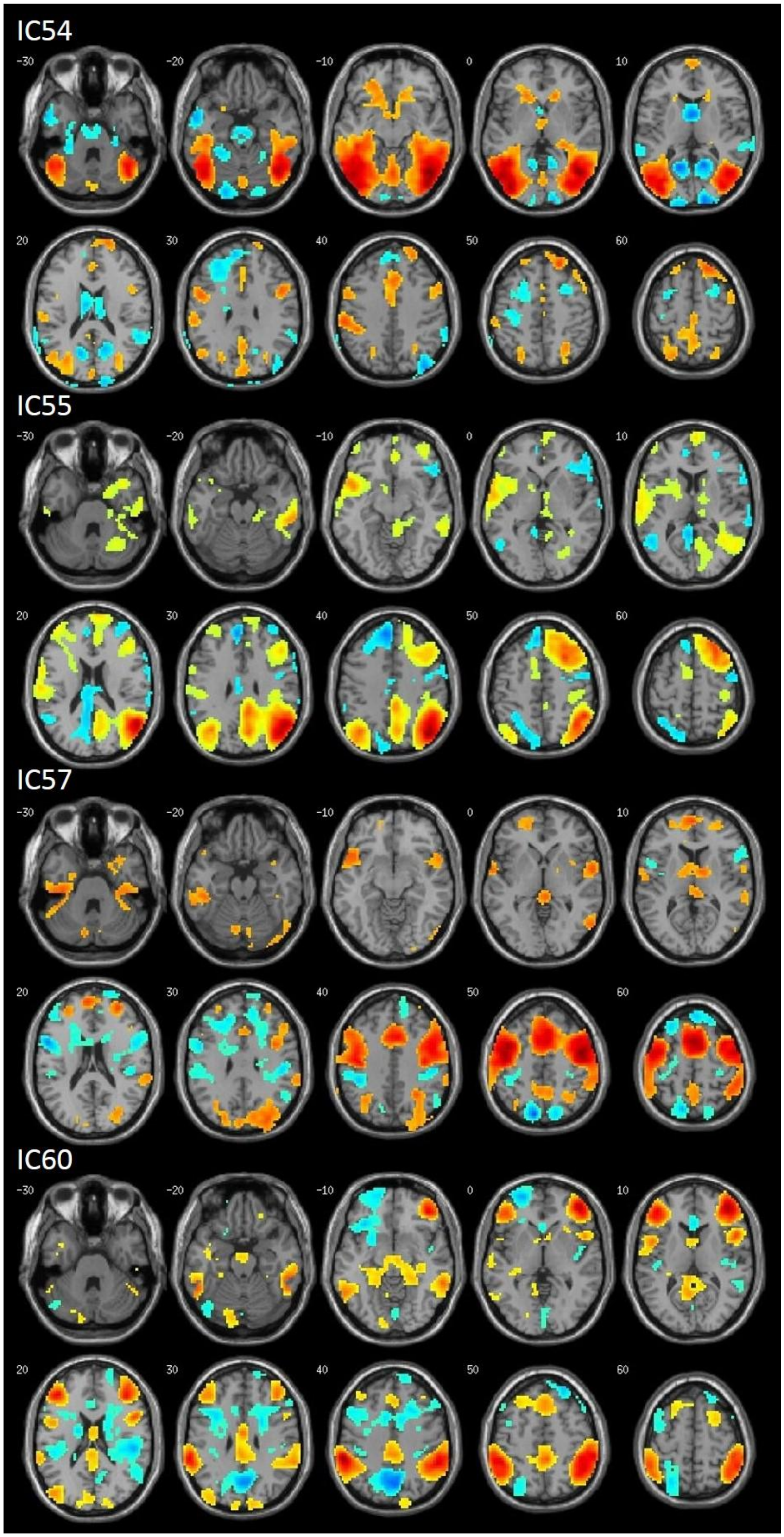
IC54
-30 -20 -10 0 10
20 30 40 50 60
IC55
-30 -20 -10 0 10
20 30 40 50 60
IC57
-30 -20 -10 0 10
20 30 40 50 60
IC60
-30 -20 -10 0 10
20 30 40 50 60

Figure S2. Spatial distributions of 32 ICs. Colors on the Montreal Neurological Institute (MNI) T1 templates demonstrate the spatial distribution of the 32 ICs labeled by their IC numbers. The red and blue colors represent positive and negative sub-networks of each IC, respectively. Only clusters surviving corrected voxel height $p < 0.05$ (FDR-corrected for whole-brain analysis) and $k > 100$ are shown. The numbers at top-left of each brain image indicates the Z coordinates in MNI space. The color bar indicates *t* values. Right side of the brain image is the right side of the brain.
