# Supplementary material for: Shared facial emotion processing functional network findings in medication-naïve major depressive disorder and healthy individuals: detection by sICA
Source: BMC Psychiatry. 2018 Apr 10;18:96. doi: 10.1186/s12888-018-1631-0 (PMC5891939; doi:10.1186/s12888-018-1631-0)
Supplement: Supplementary file 3 — Table S1. Brain regions showing task-related changes in BOLD signal assessed by SPM8. (PDF 106 kb) [file 12888_2018_1631_MOESM3_ESM.pdf]

Table S1: Positive sub-networks of 32 ICs

					MNI Coordinates		
	L/R	BA	Size (voxels)	Z-Value	X	Y	Z
**IC1**							
Precentral gyrus, IFG, Postcentral gyrus, STG	L/R	6	2226/2195	18.0/17.4	-57/63	-6/0	21/12
Cerebellum posterior lobe	L/R		452	9.7	15	-66	-33
**IC2**							
Postcentral gyrus, SMA, MFC, IPL, MFG	R	3, 4, 6	4484	18.5	42	-33	54
Cerebellum anterior/posterior lobe	L		1184	10.7	-18	-57	-36
ITG	R	20	117	7.1	57	-21	-27
IPL, Postcentral gyrus,	L	40	299	8.0	-48	-30	42
**IC3**							
IFG, MFC, SFG, MFG, ACC, Orbital gyrus	L/R	11	3686	16.6	-6	51	-18
**IC6**							
MFG, IFG, MFC	R	10, 11, 47	2438	13.2	42	30	-15
MFG, SFG	L	11	834	11.1	-36	45	-9
Precentral gyrus, MTG, IPL, STG MOG	L	40	1222	8.9	-39	-87	18
Precuneus	R	7	167	6.2	3	-66	45
MFC	R	6	141	6.2	3	-6	51
**IC7**							
Postcentral gyrus, Precentral gyrus, IPL, MCC, SMA	L	3, 4, 6, 40	4853	21.4	-39	-30	57
Cerebellum anterior/posterior lobe	R		1139	10.8	18	-57	-30
ITG	L	20	171	7.8	-54	-33	-24
IFG	R	38, 47	223	7.0	51	33	-9
Postcentral gyrus,	R	2	297	8.6	48	-24	39
**IC8**							
Precuneus, PCC, Cuneus,	L/R	7, 19, 30, 31, 39	6178	25.4	9	-45	21
SFG	L/R	8	312/176	7.9/7.4	0/24	51/39	45/45
MFC	R	6	244	9.3	3	-3	60
**IC9**							
STG, MTG, Insula,	L/R	21, 22	2619/1982	22.4/21.4	57/-60	-9/-15	0/3
SFG, MFC	L/R	6	329	6.6	0	15	57
**IC10**							
Precuneus, Cuneus	L/R	7, 18, 19	2866	23.4	15	-78	36
SFG	L	8, 9, 10, 11	112/150/126	6.6/8.5/6.5	-15/-18/-6	63/57/33	-12/33/54
Cerebellum anterior lobe	L/R		206	8.7	6	-45	-6

IPL	L	40	187	8.0	-45	-60	39
**IC11**							
IFG, MFG, MFC	L	10, 11, 47	2984	16.3	-42	48	6
MTG, STG, Postcentral gyrus, Precentral gyrus, IPL, MOG	R	19, 39, 40	1511	9.1	54	-75	18
MFC	L	8, 9	357	10.8	-3	33	39
IPL	L	7	288	9.4	-42	-66	45
**IC13**							
Precuneus, Cuneus	L/R	7, 31	3437	24.0	6	-66	51
Rectal gyrus	L/R	11, 25, 47	478	7.6	6	12	-21
MOG	L		442	9.0	-30	-69	0
MFC	L	9, 10	477	6.9	-3	54	15
MTG	L	39	150	6.2	-57	-60	6
MFG, SFG	L/R	6, 8, 9, 46	220/342	7.1/7.5	-27/33	39/48	48/42
**IC14**							
MOG, Cuneus, PCC, Precuneus	L/R	17, 18, 19	2820	23.0	18	-93	21
Cerebellum anterior/posterior lobe	L		239	7.1	-42	-51	-33
SFG	R	6, 8, 9, 10	443	8.6	18	43	27
**IC17**							
IFG, Insula, SFG	L/R	13, 38, 47	3504	24.5	33	24	-12
MFC, SFG, ACC, SMA,	L/R	6, 8, 9, 32	1942	18.4	3	33	33
MTG	R	21	516	13.3	57	-24	-12
MOG	R		222	7.2	48	-72	-9
IPL	R	40	218	8.5	51	-51	39
MFG	R	9	142	6.8	42	21	36
**IC18**							
MTG, STG, IFG, MOG, IPL, Fusiform gyrus	R	22, 37, 39, 40	5043	19.3	54	-48	9
MTG, STG, IPL,	L	40	1581	16.5	-51	-57	6
Cerebellum posterior lobe, Ligual gyrus	L		321	8.3	-15	-87	-21
MFC	L/R		212	8.7	0	42	-9
MFG	L	10	359	7.9	-36	57	3
ACC, Caudate	R		643	9.9	9	6	18
SFG	R	6	796	11.8	12	51	48
Precuneus	R	7	105	6.4	6	-60	39
MFG, Precentral gyrus	R	6	456	10.2	45	3	48
**IC19**							
MFG, SFG, MFC	L/R	10	2290	14.3	-27	57	18
SFG, SMA, MFG, MFC	L/R	6	856	8.5	0	15	60
MTG	R		193	7.3	54	-18	-18
**IC20**							
Lingual gyrus, Cuneus, MOG Cerebellum posterior lobe,	L/R	17, 18, 19	3555	23.1	3	-87	-6

Fusiform gyrus							
Precuneus	L/R	7	301	9.9	0	75	-42
SMA, MFC,	L/R	6	382	8.0	3	-6	48
**IC22**							
SFG, MFC, ACC, MFG	L/R	8, 9, 10, 32	4141	28.3	0	51	30
PCC, Precuneus	L/R		381	11.9	3	-54	15
MTG	L	39	167	7.5	-48	-72	18
**IC23**							
Cuneus, PCC, Fusiform gyrus Cerebellum posterior lobe, Lingual gyrus, Precuneus, MOG	L/R	18, 19, 30,31	6168	25.6	12	-69	-3
STG	R	22,38	212	7.8	60	9	0
MFC	L/R	9	279	7.9	0	42	18
Precentral gyrus	R	6	248	11.3	39	-18	39
Precuneus	L	7	302	7.2	-15	-63	57
**IC31**							
IPL, Postcentral gyrus, STG, Insula, IFG, Precentral gyrus	R	2, 7, 13, 40, 41	6136	23.5	57	-24	36
Cerebellum posterior lobe	R		183	7.7	18	-57	-18
MTG	L/R	37	295/257	8.9/9.1	-54/54	-63/-60	-3/-12
IFG	L	9	477	10.3	-54	9	15
SFG, MFC	L	10	681	9.6	-3	27	66
Precuneus	L	31	286	7.9	-6	-63	27
MCC	L/R	24	320	9.8	9	3	39
SFG	R	6	374	7.5	24	0	75
**IC33**							
Precuneus, SPL, PCC, MOG, lingual gyrus	L/R	7, 18, 19, 40	4905	22.4	24	-72	54
SFG, MFC	L	10	829	10.3	-9	48	39
Cerebellum posterior lobe	L		244	6.4	-21	-75	-39
Fusiform gyrus, Cerebellum posterior lobe	R	37	698	11.7	51	-54	-21
IFG, Precentral gyrus	L		643	9.6	-57	24	15
MFG	R	6	382	8.5	33	0	72
**IC34**							
IFG, MFG, Precentral gyrus, IPL, Insula, Postcentral gyrus, Precuneus, STG, MTG, Supramarginal gyrus	R	6, 9, 13, 40, 46, 47	6380	26.5	51	18	24
MOG, Fusiform gyrus, Cerebellum posterior lobe	L	18, 19	1054	9.2	-45	-78	-18
Lingual gyrus	R		151	7.7	6	-93	-9
STG	L	13	314	9.9	-39	-24	6
IFG, MFG	L		429	10.0	-45	18	27

MFC, SFG, SMA	L/R	6, 8, 9	455	12.3	3	36	45
**IC35**							
Parahippocampa gyrus, Lentiform nucleus, Putamen, IFG, Thalamus, Uncus, Pons, MFG, Caudate, Insula	L/R	47	6424	13.0	24	12	-15
**IC36**							
IFG, Insula, STG, STG, MFG, Lentiform nucleus, Putamen, Precentral gyrus, IPL, ACC, SFG, MFC	L/R	6, 9,10, 13, 22, 44, 47	6550	18.4	36	12	-3
MCC, MFC, SMA,	L/R	8, 24, 32	1940	12.8	-6	6	45
Cerebellum posterior lobe	L/R		154/279	6.7/7.6	-39/42	-75/-63	-27/-27
ITG, Fusiform gyrus	R	20	352	8.6	42	-12	-30
MTG	L	39	159	6.0	-42	-75	24
IPL	R	40	200	9.1	60	-33	27
Precentral gyrus	L		177	7.4	-45	-9	57
**IC37**							
IFG, MTG, STG, ITG	L	21, 47	2938	22.1	-54	21	9
IFG, Precentral gyrus, STG	R	47	1400	17.2	45	30	-15
MOG	R	19	353	7.0	30	-81	6
Cerebellum posterior lobe	R		284	6.4	30	-72	-36
SFG	L	10	230	10.1	-18	57	27
SFG, SMA,	L/R	6, 10	1372	13.6	-9	12	66
MCC	L/R		306	9.2	0	-12	39
IPL	L	40	249	8.1	-45	-39	57
**IC40**							
MFG, IFG, IPL, MFC, Precentral gyrus, SFG, Postcentral gyrus, Insula, Precuneus	L	6, 8, 9, 40, 46	6483	25.7	-48	24	27
SFG	R	10	175	9.4	27	57	3
MFG, IFG	R	9, 46	955	17.0	54	33	27
MOG	R	19	372	8.3	57	-69	15
**IC44**							
MFC, ACC, IFG, SFG, Insula	L/R	10	4698	22.7	-3	42	3
MCC	L/R		452	9.3	0	-12	30
MFG	R		536	7.6	42	0	60
Cerebellum posterior lobe	L		399	9.5	-27	-33	-21
Parahippocampal gyrus	R		295	7.8	24	-18	-30
**IC45**							
MFG, SFG, MFC, SMA, IFG, ACC, Precentral gyrus, Precuneus	L	6, 7, 8, 9, 10, 32	6551	21.0	-30	51	24
MTG	R	19	716	9.0	51	-78	24
IFG	R		190	7.4	48	33	15

Supramarginal gyrus, IPL	L	40	404	9.2	-57	-51	33
Precuneus	R		175	6.6	15	-69	33
Postcentral gyrus	R		243	6.4	39	-24	45
**IC50**							
MFG, SFG, MFC, MCC, IFG, ACC, STG, SMA, Insula	L/R	6, 8, 9, 10, 32, 46	6377	21.4	39	42	33
MTG, MOG	L	19	1053	10.6	-48	-84	9
Lingual gyrus	R		188	9.0	12	-96	-15
IPL, Supramarginal gyrus	R	40	428	9.7	63	-42	36
Precuneus	R	7	531	7.5	6	-60	48
**IC52**							
IPL, Precuneus, SPL, Angular gyrus	L	7, 40	2692	19.8	-33	-63	48
IPL	R	40	692	16.0	45	-63	54
IFG, Precentral gyrus, STG	R		540	9.2	54	24	-6
SFG	R		119	7.3	15	48	36
STG	R		223	8.6	60	-27	12
**IC54**							
MOG, Fusiform gyrus, MTG, Cerebellum posterior lobe, ITG, Parahippocampa gyrus	L/R	18, 19, 37	3781/3213	13.8/15.3	-36/45	-81/-66	0/-6
SFG	L/R	9	925	10.2	21	42	54
IPL, Postcentral gyrus	L	40	340	8.6	-57	-27	42
MFC, MCC	L/R	32	663	8.1	3	21	42
**IC55**							
MTG, IPL, PCC, STG, Cuneus, Supramarginal gyrus, ITG, Angular gyrus	R	7, 19, 20, 31, 39, 40	5797	26.2	45	-72	36
MFG, SFG, MFC	R	6, 8, 9, 10	2687	17.7	30	30	57
STG, MFG, IFG, Insula, Precentral gyrus, Postcentral gyrus	L	22	2089	13.1	-48	9	-9
Angular gyrus, Precuneus	L	39	753	15.8	-39	-69	33
SFG, MFC	L	6	345	10.8	-6	3	75
**IC57**							
SFG, MFC, Precentral gyrus, SMA, MCC, Cuneus, Precuneus, IPL, MTG	L/R	6, 8, 19, 32, 40	5609	14.9	-3	6	66
STG	L/R		222/219	8.0/7.9	-48/60	15/0	-12/0
MFC	L/R		434	10.9	-3	57	15
Precuneus	L/R	7	418	7.9	0	-45	72
MFG, Precentral gyrus, Postcentral gyrus	L	6	1742	13.4	-45	-9	51
**IC60**							
MFG, SFG, MFC,	L	8	1407	16.8	-45	39	15
MTG	L/R		336/401	10.5/11.2	-54/57	-51/-48	-21/-18

MFG, IFG	R	10, 46	1208	20.1	42	48	6
PCC, MCC	L/R		1252	12.3	3	-36	48
IPL, Supramarginal gyrus	L/R	40	1344/1574	19.1/16.5	-60/54	-39/-42	39/42

The MNI (Montreal Neurological Institute) coordinates show the coordinates of the peak voxels. Clusters with k < 100 are not listed. ACC: anterior cingulate gyrus; SFG: superior frontal gyrus; MFG: middle frontal gyrus; IFG: inferior frontal gyrus; MFC: medial frontal cortex; MTG: middle temporal gyrus; STG: superior temporal gyrus; MTG: middle temporal gyrus; ITG: inferior temporal gyrus; PCC: posterior cingulate gyrus; SPL: superior parietal lobule; IPL: inferior parietal lobule; SMA: supplementary motor area; MCC: mid-cingulate cortex; MOG: middle occipital gyrus.
