# Supplementary material for: Shared facial emotion processing functional network findings in medication-naïve major depressive disorder and healthy individuals: detection by sICA
Source: BMC Psychiatry. 2018 Apr 10;18:96. doi: 10.1186/s12888-018-1631-0 (PMC5891939; doi:10.1186/s12888-018-1631-0)
Supplement: Supplementary file 4 — Table S2. Betaweights at each task condition and related p values. (PDF 99 kb) [file 12888_2018_1631_MOESM4_ESM.pdf]

# Table S2 Beta weights of all ICs at each task condition and related *p* values

| IC | | 1 | 2 | 3 | 6 | 7 | 8 | 9 | 10 | 11 | 13 | 14 | 17 | 18 | 19 | 20 | 22 |
|---|---|---|---|---|---|---|---|---|---|---|---|---|---|---|---|---|---|
| **Mean beta weight values** | | | | | | | | | | | | | | | | | |
| **F** | **1** | 0.143 | -0.381 | -0.348 | -0.050 | -0.180 | 0.194 | 0.426 | -0.099 | 0.070 | -0.191 | 0.058 | 0.011 | 0.057 | -0.175 | -0.247 | 0.106 |
| | | 0.470 | 1.463 | 1.221 | 1.273 | 0.854 | 0.829 | 0.801 | 1.420 | 1.147 | 1.711 | 0.887 | 0.560 | 0.375 | 0.848 | 0.792 | 0.880 |
| | **2** | 0.449 | -0.135 | -0.434 | 0.062 | -0.135 | -0.085 | 0.586 | -0.144 | -0.111 | -0.733 | -0.341 | -0.043 | 0.025 | 0.384 | -0.329 | 0.207 |
| | | 0.585 | 0.956 | 1.144 | 2.215 | 0.979 | 0.799 | 1.000 | 1.567 | 0.738 | 1.643 | 0.961 | 0.608 | 0.583 | 0.812 | 1.158 | 1.035 |
| | **all** | 0.287 | -0.265 | -0.388 | 0.003 | -0.159 | 0.062 | 0.501 | -0.120 | -0.015 | -0.446 | -0.130 | -0.014 | 0.042 | 0.088 | -0.285 | 0.153 |
| | | 0.545 | 1.248 | 1.177 | 1.766 | 0.908 | 0.821 | 0.897 | 1.480 | 0.974 | 1.689 | 0.937 | 0.579 | 0.481 | 0.872 | 0.974 | 0.950 |
| **H** | **1** | 0.133 | -0.355 | 0.401 | 0.127 | -0.320 | -0.175 | -0.016 | -0.152 | -0.136 | 0.231 | -0.127 | -0.255 | -0.133 | 0.019 | -0.321 | -0.023 |
| | | 0.673 | 0.818 | 1.017 | 1.362 | 0.747 | 1.069 | 0.731 | 1.517 | 0.671 | 1.608 | 1.070 | 0.548 | 0.577 | 1.130 | 0.755 | 1.012 |
| | **2** | -0.122 | -0.189 | 0.386 | -0.133 | -0.225 | 0.229 | -0.143 | -0.188 | 0.185 | 0.200 | 0.142 | -0.126 | -0.045 | -0.204 | -0.130 | 0.001 |
| | | 0.541 | 0.771 | 1.030 | 2.459 | 0.622 | 1.084 | 0.874 | 1.296 | 1.466 | 2.041 | 1.281 | 1.089 | 0.606 | 1.689 | 1.071 | 1.055 |
| | **all** | 0.013 | -0.277 | 0.394 | 0.004 | -0.275 | 0.015 | -0.075 | -0.169 | 0.015 | 0.217 | 0.000 | -0.194 | -0.092 | -0.086 | -0.231 | -0.012 |
| | | 0.624 | 0.795 | 1.016 | 1.946 | 0.687 | 1.087 | 0.798 | 1.407 | 1.120 | 1.811 | 1.173 | 0.842 | 0.588 | 1.414 | 0.916 | 1.025 |
| **N** | **1** | -0.150 | 0.237 | -0.251 | -0.277 | 0.007 | 0.354 | -0.122 | 0.148 | 0.131 | -0.358 | 0.158 | 0.165 | 0.171 | 0.138 | 0.191 | 0.170 |
| | | 0.495 | 0.649 | 1.172 | 0.929 | 0.793 | 1.111 | 0.632 | 1.283 | 0.712 | 1.843 | 0.824 | 0.551 | 0.504 | 1.062 | 0.819 | 0.674 |
| | **2** | -0.093 | -0.014 | 0.086 | -0.007 | -0.109 | 0.167 | -0.074 | 0.302 | -0.150 | 0.410 | 0.102 | 0.030 | 0.187 | -0.140 | 0.085 | 0.086 |
| | | 0.780 | 0.871 | 1.479 | 2.480 | 0.952 | 0.796 | 1.146 | 1.223 | 1.217 | 2.241 | 1.220 | 0.848 | 0.629 | 1.879 | 0.716 | 0.634 |
| | **all** | -0.124 | 0.119 | -0.092 | -0.150 | -0.047 | 0.266 | -0.100 | 0.221 | -0.001 | 0.004 | 0.132 | 0.102 | 0.179 | 0.007 | 0.141 | 0.130 |
| | | 0.641 | 0.766 | 1.326 | 1.820 | 0.867 | 0.973 | 0.904 | 1.248 | 0.985 | 2.061 | 1.021 | 0.704 | 0.562 | 1.498 | 0.769 | 0.652 |
| ***P* values of group difference, condition difference, and their interaction** | | | | | | | | | | | | | | | | | |
| **Group** | | .436 | .580 | .511 | .750 | .915 | .793 | .691 | .869 | .589 | .680 | .395 | .720 | .519 | .811 | .987 | .847 |
| **Con** | | .000* | .030 | .004* | .907 | .260 | .392 | .000* | .228 | .979 | .163 | .306 | .255 | .121 | .515 | .029 | .684 |
| **Inter** | | .065 | .353 | .610 | .769 | .792 | .155 | .708 | .913 | .286 | .200 | .291 | .644 | .864 | .206 | .644 | .872 |
| **Continued** | | | | | | | | | | | | | | | | | |
| **IC** | | **23** | **31** | **33** | **34** | **35** | **36** | **37** | **40** | **44** | **45** | **50** | **52** | **54** | **55** | **57** | **60** |
| **Mean beta weight values** | | | | | | | | | | | | | | | | | |

| | | | | | | | | | | | | | | | | | |
|---|---|---|---|---|---|---|---|---|---|---|---|---|---|---|---|---|---|
| **F** | **1** | 0.085 | -0.173 | -0.327 | -0.285 | 0.051 | -0.022 | 0.098 | -0.182 | 0.063 | 0.152 | -0.371 | -0.186 | -0.207 | 0.011 | -0.162 | -0.306 |
| | | 0.734 | 0.802 | 1.344 | 0.746 | 0.445 | 0.539 | 0.597 | 0.700 | 1.068 | 0.881 | 1.133 | 1.176 | 0.637 | 0.905 | 0.693 | 0.952 |
| | **2** | -0.026 | 0.000 | -0.273 | -0.167 | 0.003 | 0.081 | -0.002 | 0.052 | -0.056 | 0.312 | -0.214 | 0.363 | -0.203 | -0.022 | -0.111 | -0.080 |
| | | 1.006 | 0.827 | 1.537 | 0.539 | 0.319 | 0.906 | 0.596 | 0.518 | 0.895 | 1.071 | 0.805 | 1.092 | 0.659 | 0.682 | 0.700 | 0.580 |
| | **all** | 0.033 | -0.091 | -0.301 | -0.230 | 0.028 | 0.027 | 0.051 | -0.072 | 0.007 | 0.227 | -0.297 | 0.073 | -0.205 | -0.004 | -0.138 | -0.200 |
| | | 0.867 | 0.812 | 1.428 | 0.655 | 0.389 | 0.731 | 0.594 | 0.627 | 0.985 | 0.971 | 0.988 | 1.162 | 0.643 | 0.802 | 0.691 | 0.801 |
| **H** | **1** | -0.018 | -0.129 | -0.224 | -0.308 | -0.030 | -0.046 | 0.053 | -0.097 | -0.101 | 0.071 | -0.327 | -0.228 | -0.278 | -0.201 | -0.142 | -0.182 |
| | | 0.859 | 0.688 | 1.300 | 0.908 | 0.324 | 0.424 | 0.621 | 0.564 | 0.875 | 0.813 | 1.294 | 0.846 | 0.579 | 0.936 | 0.602 | 1.098 |
| | **2** | 0.125 | -0.255 | -0.227 | -0.274 | -0.057 | -0.258 | 0.062 | -0.220 | 0.166 | 0.022 | -0.103 | -0.067 | -0.056 | 0.070 | -0.299 | -0.077 |
| | | 0.840 | 0.860 | 1.418 | 0.831 | 0.350 | 0.640 | 0.427 | 1.027 | 0.785 | 1.076 | 1.118 | 1.727 | 0.694 | 0.840 | 0.769 | 1.036 |
| | **all** | 0.049 | -0.188 | -0.226 | -0.292 | -0.042 | -0.146 | 0.057 | -0.155 | 0.024 | 0.048 | -0.222 | -0.152 | -0.173 | -0.074 | -0.216 | -0.133 |
| | | 0.847 | 0.770 | 1.346 | 0.866 | 0.334 | 0.543 | 0.534 | 0.811 | 0.838 | 0.939 | 1.211 | 1.327 | 0.641 | 0.896 | 0.685 | 1.062 |
| **N** | **1** | 0.085 | 0.056 | -0.085 | 0.253 | 0.065 | 0.089 | -0.016 | 0.179 | -0.034 | -0.004 | 0.192 | 0.113 | -0.016 | 0.382 | 0.108 | -0.071 |
| | | 0.847 | 0.670 | 0.960 | 0.622 | 0.261 | 0.542 | 0.461 | 0.694 | 0.722 | 0.649 | 1.085 | 1.145 | 0.897 | 0.679 | 0.567 | 0.760 |
| | **2** | -0.049 | -0.038 | 0.443 | 0.111 | 0.056 | -0.023 | 0.072 | 0.116 | 0.015 | 0.142 | 0.166 | -0.254 | -0.096 | 0.150 | 0.207 | -0.126 |
| | | 1.077 | 0.899 | 1.291 | 0.750 | 0.386 | 0.652 | 0.657 | 1.156 | 0.743 | 1.008 | 0.964 | 1.909 | 1.066 | 0.657 | 0.706 | 0.830 |
| | **all** | 0.022 | 0.012 | 0.163 | 0.186 | 0.061 | 0.036 | 0.025 | 0.149 | -0.011 | 0.065 | 0.180 | -0.059 | -0.054 | 0.273 | 0.154 | -0.097 |
| | | 0.957 | 0.781 | 1.150 | 0.684 | 0.323 | 0.595 | 0.559 | 0.933 | 0.727 | 0.834 | 1.022 | 1.551 | 0.973 | 0.674 | 0.634 | 0.788 |
| ***P* values of group difference, condition difference, and their interaction** | | | | | | | | | | | | | | | | | |
| **Group** | | .610 | .779 | .071 | .961 | .379 | .171 | .985 | .756 | .320 | .337 | .140 | .213 | .972 | .463 | .971 | .390 |
| **Con** | | .983 | .468 | .067 | .002* | .297 | .154 | .960 | .283 | .965 | .570 | .040 | .521 | .001* | .920 | .008 | .751 |
| **Inter** | | .693 | .588 | .538 | .660 | .959 | .432 | .687 | .503 | .532 | .806 | .837 | .246 | .208 | .567 | .586 | .695 |

The IC row shows the IC numbers.

F: Fearful; H: happy; N: neutral; Group: group difference; Con: condition difference; Inter: group and condition interaction. In each condition, 1: health control; 2: MDD; all: all the subjects. In each group, the upper row shows mean beta weight values, the lower row shows the standard deviations.

*: IC that survived after FDR correction with $p < 0.05$.
